# Expert Consensus on the Diagnosis and Treatment of FGFR Gene-Altered Solid Tumors

**DOI:** 10.1055/s-0044-1790230

**Published:** 2024-09-16

**Authors:** Chunwei Xu, Bin Lian, Juanjuan Ou, Qian Wang, Wenxian Wang, Ke Wang, Dong Wang, Zhengbo Song, Aijun Liu, Jinpu Yu, Wenzhao Zhong, Zhijie Wang, Yongchang Zhang, Jingjing Liu, Shirong Zhang, Xiuyu Cai, Anwen Liu, Wen Li, Lili Mao, Ping Zhan, Hongbing Liu, Tangfeng Lv, Liyun Miao, Lingfeng Min, Yu Chen, Jingping Yuan, Feng Wang, Zhansheng Jiang, Gen Lin, Long Huang, Xingxiang Pu, Rongbo Lin, Weifeng Liu, Chuangzhou Rao, Dongqing Lv, Zongyang Yu, Xiaoyan Li, Chuanhao Tang, Chengzhi Zhou, Junping Zhang, Junli Xue, Hui Guo, Qian Chu, Rui Meng, Jingxun Wu, Rui Zhang, Jin Zhou, Zhengfei Zhu, Yongheng Li, Hong Qiu, Fan Xia, Yuanyuan Lu, Xiaofeng Chen, Rui Ge, Enyong Dai, Yu Han, Weiwei Pan, Fei Pang, Jintao Huang, Kai Wang, Fan Wu, Bingwei Xu, Liping Wang, Youcai Zhu, Li Lin, Yanru Xie, Xinqing Lin, Jing Cai, Ling Xu, Jisheng Li, Xiaodong Jiao, Kainan Li, Jia Wei, Huijing Feng, Lin Wang, Yingying Du, Wang Yao, Xuefei Shi, Xiaomin Niu, Dongmei Yuan, Yanwen Yao, Jianhui Huang, Yue Feng, Yinbin Zhang, Pingli Sun, Hong Wang, Mingxiang Ye, Zhaofeng Wang, Yue Hao, Zhen Wang, Bin Wan, Donglai Lv, Zhanqiang Zhai, Shengjie Yang, Jing Kang, Jiatao Zhang, Chao Zhang, Lin Shi, Yina Wang, Bihui Li, Zhang Zhang, Zhongwu Li, Zhefeng Liu, Nong Yang, Lin Wu, Huijuan Wang, Gu Jin, Guansong Wang, Jiandong Wang, Meiyu Fang, Yong Fang, Yuan Li, Xiaojia Wang, Jing Chen, Yiping Zhang, Xixu Zhu, Yi Shen, Shenglin Ma, Biyun Wang, Lu Si, Yuanzhi Lu, Ziming Li, Wenfeng Fang, Yong Song

**Affiliations:** 1 Department of Scientific Research, Institute of Cancer and Basic Medicine (ICBM), Chinese Academy of Sciences, Hangzhou Zhejiang 310022, People's Republic of China; 2 Department of Respiratory Medicine, Affiliated Jinling Hospital, Medical School of Nanjing University, Nanjing, Jiangsu, People's Republic of China; 3 Key Laboratory of Carcinogenesis and Translational Research (Ministry of Education/Beijing), Department of Melanoma and Sarcoma, Peking University Cancer Hospital and Institute, Beijing, People's Republic of China; 4 Department of Oncology and Southwest Cancer Center, Southwest Hospital, Third Military Medical University (Army Medical University), Chongqing, People's Republic of China; 5 Department of Respiratory Medicine, Affiliated Hospital of Nanjing University of Chinese Medicine, Jiangsu Province Hospital of Chinese Medicine, Nanjing, Jiangsu, People's Republic of China; 6 Department of Chemotherapy, Chinese Academy of Sciences University Cancer Hospital (Zhejiang Cancer Hospital), Hangzhou, Zhejiang, People's Republic of China; 7 National Health Commission Key Laboratory of Nuclear Medicine, Jiangsu Key Laboratory of Molecular Nuclear Medicine, Jiangsu Institute of Nuclear Medicine, Wuxi Jiangsu, People's Republic of China; 8 Department of Radiopharmaceuticals, School of Pharmacy, Nanjing Medical University, Nanjing, Jiangsu, People's Republic of China; 9 Senior Department of Pathology, the 7th Medical Center of PLA General Hospital, Beijing, People's Republic of China; 10 Department of Cancer Molecular Diagnostics Core, Tianjin Medical University Cancer Institute and Hospital, Tianjin, People's Republic of China; 11 Department of Guangdong Lung Cancer Institute, Guangdong Provincial Laboratory of Translational Medicine in Lung Cancer, Guangdong Provincial People's Hospital, Guangdong Academy of Medical Sciences, School of Medicine, Guangzhou Guangdong, People's Republic of China; 12 State Key Laboratory of Molecular Oncology, Department of Medical Oncology, National Cancer Center/National Clinical Research Center for Cancer/Cancer Hospital, Chinese Academy of Medical Sciences and Peking Union Medical College, Beijing, People's Republic of China; 13 Department of Medical Oncology, Lung Cancer and Gastrointestinal Unit, Hunan Cancer Hospital/The Affiliated Cancer Hospital of Xiangya School of Medicine, Central South University, Changsha, Hunan, People's Republic of China; 14 Department of Thoracic Cancer, Jilin Cancer Hospital, Changchun, Jilin, People's Republic of China; 15 Department of Translational Medicine Research Center, Key Laboratory of Clinical Cancer Pharmacology and Toxicology Research of Zhejiang Province, Affiliated Hangzhou First People's Hospital, Cancer Center, Zhejiang University School of Medicine, Hangzhou Zhejiang, People's Republic of China; 16 Department of VIP Inpatient, Sun Yet-Sen University Cancer Center, State Key Laboratory of Oncology in South China, Collaborative Innovation Center for Cancer Medicine, Guangzhou, Guangdong, People's Republic of China; 17 Department of Oncology, Second Affiliated Hospital of Nanchang University, Nanchang, Jiangxi, People's Republic of China; 18 Key Laboratory of Respiratory Disease of Zhejiang Province, Department of Respiratory and Critical Care Medicine, Second Affiliated Hospital of Zhejiang University School of Medicine, Cancer Center, Zhejiang University, Hangzhou, Zhejiang, People's Republic of China; 19 Department of Respiratory Medicine, Affiliated Drum Tower Hospital, Medical School of Nanjing University, Nanjing, Jiangsu, People's Republic of China; 20 Department of Respiratory Medicine, Clinical Medical School of Yangzhou University, Subei People's Hospital of Jiangsu Province, Yangzhou, Jiangsu, People's Republic of China; 21 Department of Medical Oncology, Fujian Medical University Cancer Hospital and Fujian Cancer Hospital, Fuzhou, Fujian, People's Republic of China; 22 Department of Pathology, Renmin Hospital of Wuhan University, Wuhan, Hubei, People's Republic of China; 23 Department of Internal Medicine, Cancer Center of PLA, Qinhuai Medical Area, Affiliated Jinling Hospital, Medical School of Nanjing University, Nanjing, Jiangsu, People's Republic of China; 24 Derpartment of Integrative Oncology, Tianjin Medical University Cancer Institute and Hospital, Tianjin, People's Republic of China; 25 Department of Medical Oncology, Lung Cancer and Hunan Cancer Hospital/The Affiliated Cancer Hospital of Xiangya School of Medicine, Central South University, Changsha, Hunan, People's Republic of China; 26 Department of Orthopaedic Oncology Surgery, Beijing Ji Shui Tan Hospital, Peking University, Beijing, People's Republic of China; 27 Department of Radiotherapy and Chemotherapy, Hwamei Hospital, University of Chinese Academy of Sciences, Ningbo, Zhejiang, People's Republic of China; 28 Department of Pulmonary Medicine, Taizhou Hospital of Wenzhou Medical University, Taizhou, Zhejiang, People's Republic of China; 29 Department of Respiratory Medicine, the 900th Hospital of the Joint Logistics Team (the Former Fuzhou General Hospital), Fujian Medical University, Fuzhou, Fujian, People's Republic of China; 30 Department of Oncology, Beijing Tiantan Hospital, Capital Medical University, Beijing, People's Republic of China; 31 Department of Medical Oncology, Peking University International Hospital, Beijing, People's Republic of China; 32 Department of State Key Laboratory of Respiratory Disease, National Clinical Research Center for Respiratory Disease; Guangzhou Institute of Respiratory Health, The First Affiliated Hospital of Guangzhou Medical University (The First Affiliated Hospital of Guangzhou Medical University), Guangzhou Guangdong, People's Republic of China; 33 Department of Thoracic Oncology, Shanxi Academy of Medical Sciences, Shanxi Bethune Hospital, Taiyuan, Shanxi, People's Republic of China; 34 Department of Oncology, Shanghai East Hospital, School of Medicine, Tongji University, Shanghai, People's Republic of China; 35 Department of Medical Oncology, The First Affiliated Hospital of Xi'an Jiaotong University, Xi'an, Shaanxi, People's Republic of China; 36 Department of Oncology, Tongji Hospital of Tongji Medical College, Huazhong University of Science and Technology, Wuhan, Hubei, People's Republic of China; 37 Department of Cancer Center, Union Hospital, Tongji Medical College, Huazhong University of Science and Technology, Wuhan Hubei, People's Republic of China; 38 Department of Medical Oncology, the First Affiliated Hospital of Medicine, Xiamen University, Xiamen, Fujian, People's Republic of China; 39 Department of Medical Oncology, Cancer Hospital of China Medical University, Shenyang, Liaoning, People's Republic of China; 40 Department of Medical Oncology, Sichuan Cancer Hospital and Institute, Sichuan Cancer Center, School of Medicine, University of Electronic Science and Technology, Chengdu, Sichuan, People's Republic of China; 41 Department of Radiation Oncology, Fudan University Shanghai Cancer Center, Shanghai, People's Republic of China; 42 Key Laboratory of Carcinogenesis and Translational Research (Ministry of Education/Beijing), Department of Radiation Oncology, Peking University Cancer Hospital and Institute, Beijing, People's Republic of China; 43 Department of State Key Laboratory of Cancer Biology, National Clinical Research Center for Digestive Diseases and Xijing Hospital of Digestive Diseases, Fourth Military Medical University, Xi'an Shaanxi, People's Republic of China; 44 Department of Oncology, Jiangsu Province Hospital and Nanjing Medical University First Affiliated Hospital, Nanjing, Jiangsu, People's Republic of China; 45 Department of General Surgery, Huadong Hospital Affiliated to Fudan University, Shanghai, People's Republic of China; 46 Department of Oncology and Hematology, China-Japan Union Hospital of Jilin University, Changchun, Jilin, People's Republic of China; 47 Department of Gastrointestinal Oncology, Harbin Medical University Cancer Hospital, Harbin, Heilongjiang, People's Republic of China; 48 Department of Cell Biology, College of Medicine, Jiaxing University, Jiaxing, Zhejiang, People's Republic of China; 49 Department of Medical, Shanghai OrigiMed Co, Ltd, Shanghai, People's Republic of China; 50 Department of Medical, Menarini Silicon Biosystems Spa, Shanghai, People's Republic of China; 51 Department of Biotherapy, Cancer Institute, First Affiliated Hospital of China Medical University, Shenyang, People's Republic of China; 52 Department of Oncology, Baotou Cancer Hospital, Baotou Inner Mongolia, People's Republic of China; 53 Department of Thoracic Disease Diagnosis and Treatment Center, Zhejiang Rongjun Hospital, The Third Affiliated Hospital of Jiaxing University, Jiaxing, Zhejiang, People's Republic of China; 54 Department of Oncology, Lishui Municipal Central Hospital, Lishui, Zhejiang, People's Republic of China; 55 Department of Interventional Pulmonary Diseases, Anhui Chest Hospital, Hefei, Anhui, People's Republic of China; 56 Department of Medical Oncology, Qilu Hospital, Cheeloo College of Medicine, Shandong University, Jinan, Shandong, People's Republic of China; 57 Department of Medical Oncology, Shanghai Changzheng Hospital, Naval Medical University, Shanghai, People's Republic of China; 58 Department of Oncology, Shandong Provincial Third Hospital, Cheeloo College of Medicine, Shandong University, Jinan, Shandong, People's Republic of China; 59 Department of the Comprehensive Cancer Center, Affiliated Drum Tower Hospital, Medical School of Nanjing University, Nanjing, Jiangsu, People's Republic of China; 60 Department of Pathology, Shanxi Academy of Medical Sciences, Shanxi Bethune Hospital, Taiyuan, Shanxi, People's Republic of China; 61 Department of Oncology, The First Affiliated Hospital of Anhui Medical University, Hefei, Anhui, People's Republic of China; 62 Department of Interventional Oncology, The First Affiliated Hospital, Sun Yat-sen University, Guangzhou, Guangdong, People's Republic of China; 63 Department of Respiratory Medicine, Huzhou Hospital, Zhejiang University School of Medicine, Huzhou, Zhejiang, People's Republic of China; 64 Department of Shanghai Lung Cancer Center, Shanghai Chest Hospital, Shanghai Jiao Tong University, Shanghai, People's Republic of China; 65 Department of Gynecologic Radiation Oncology, Chinese Academy of Sciences University Cancer Hospital (Zhejiang Cancer Hospital), Hangzhou, Zhejiang, People's Republic of China; 66 Department of Oncology, the Second Affiliated Hospital of Medical College, Xi′an Jiaotong University, Xi'an, Shaanxi, People's Republic of China; 67 Department of Pathology, The Second Hospital of Jilin University, Changchun, Jilin, People's Republic of China; 68 Senior Department of Oncology, The 5th Medical Center of PLA General Hospital, Beijing, People's Republic of China; 69 Department of Radiation Oncology, Affiliated Jinling Hospital, Medical School of Nanjing University, Nanjing, Jiangsu, People's Republic of China; 70 Department of Respiratory Medicine, The Affiliated Jiangning Hospital of Nanjing Medical University, Nanjing, Jiangsu, People's Republic of China; 71 Department of Clinical Oncology, the 901 Hospital of Joint Logistics Support Force of People Liberation Army, Hefei, Anhui, People's Republic of China; 72 Department of Thoracic Surgery, Chuxiong Yi Autonomous Prefecture People's Hospital, Chuxiong, Yunnan, People's Republic of China; 73 Department of Respiratory Medicine, Zhongshan Hospital, Fudan University, Shanghai, People's Republic of China; 74 Department of Oncology, The First Affiliated Hospital, College of Medicine, Zhejiang University, Hangzhou, Zhejiang, People's Republic of China; 75 Department of Oncology, The Second Affiliated Hospital of Guilin Medical University, Guilin, Guangxi, People's Republic of China; 76 Department of International Cooperative Laboratory of Traditional Chinese Medicine Modernization and Innovative Drug Discovery of Chinese Ministry of Education (MOE), Guangzhou City Key Laboratory of Precision Chemical Drug Development, School of Pharmacy, Jinan University, Guangzhou, Guangdong, People's Republic of China; 77 Key Laboratory of Carcinogenesis and Translational Research (Ministry of Education/Beijing), Department of Pathology, Peking University Cancer Hospital and Institute, Beijing, People's Republic of China; 78 Department of Internal Medicine, the Affiliated Cancer Hospital of Zhengzhou University, Henan Cancer Hospital, Zhengzhou, Henan, , People's Republic of China; 79 Department of Bone and Soft-tissue Surgery, Chinese Academy of Sciences University Cancer Hospital (Zhejiang Cancer Hospital), Hangzhou, Zhejiang, People's Republic of China; 80 Department of Institute of Respiratory Diseases, Xinqiao Hospital, Third Military Medical University, Chongqing, People's Republic of China; 81 Department of Pathology, Affiliated Jinling Hospital, Medical School of Nanjing University, Nanjing, Jiangsu, People's Republic of China; 82 Department of Medical Oncology, Sir Run Run Shaw Hospital, Zhejiang University, Hangzhou, Zhejiang, People's Republic of China; 83 Department of Pathology, Fudan University Shanghai Cancer Center, Shanghai, People's Republic of China; 84 Department of Thoracic Surgery, Affiliated Jinling Hospital, Medical School of Nanjing University, Nanjing, Jiangsu, People's Republic of China; 85 Department of Oncology, Key Laboratory of Clinical Cancer Pharmacology and Toxicology Research of Zhejiang Province, Affiliated Hangzhou Cancer Hospital, Cancer Center, Zhejiang University School of Medicine, Hangzhou, Zhejiang, People's Republic of China; 86 Department of Breast Cancer and Urological Medical Oncology, Fudan University Shanghai Cancer Center, Department of Oncology, Shanghai Medical College, Fudan University, Shanghai, People's Republic of China; 87 Department of Clinical Pathology, the First Affiliated Hospital of Jinan University, Guangzhou, Guangdong, People's Republic of China; 88 Department of Medical Oncology, Sun Yat-sen University Cancer Center, State Key Laboratory of Oncology in South China, Collaborative Innovation Center for Cancer Medicine, Guangzhou, Guangdong, People's Republic of China

**Keywords:** solid tumors, tyrosine receptor kinase, precision medicine, targeted therapy

## Abstract

The fibroblast growth factor receptor (FGFR) is a crucial receptor tyrosine kinase involved in essential biological processes, including growth, development, and tissue repair. However, FGFR gene mutations, including amplification, fusion, and mutation, can disrupt epigenetics, transcriptional regulation, and tumor microenvironment interactions, leading to cancer development. Targeting these kinase mutations with small molecule drugs or antibodies has shown clinical benefits. For example, erdafitinib is approved for treating locally advanced or metastatic urothelial cancer patients with FGFR2/FGFR3 mutations, and pemigatinib is approved for treating cholangiocarcinoma with FGFR2 fusion/rearrangement. Effective screening of FGFR variant patients is crucial for the clinical application of FGFR inhibitors. Various detection methods, such as polymerase chain reaction, next-generation sequencing, fluorescence in situ hybridization, and immunohistochemistry, are available, and their selection should be based on diagnostic and treatment decision-making needs. Our developed expert consensus aims to standardize the diagnosis and treatment process for FGFR gene mutations and facilitate the practical application of FGFR inhibitors in clinical practice.

## Introductions

**Fig. 1:**
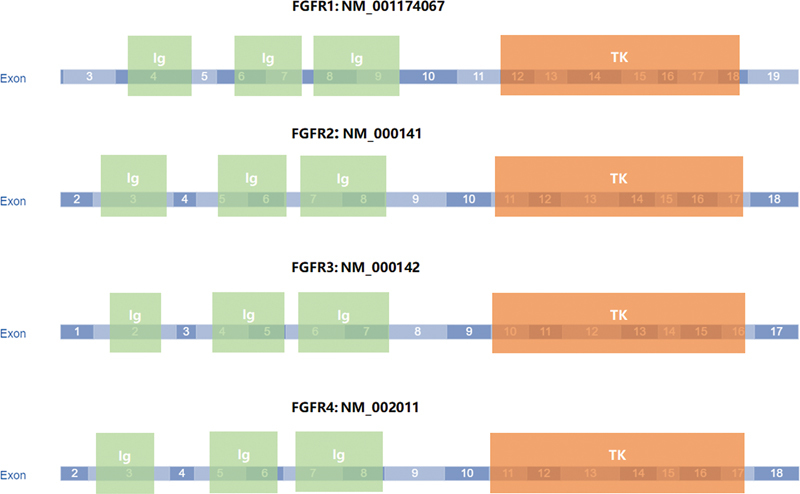
The structure of FGFR1/2/3/4 gene coding region. Ig, Immunoglobulin domain; TK, Tyrosine kinase domain.

Fibroblast growth factor receptors (FGFR) are a subfamily of highly conserved receptor tyrosine kinases, including FGFR1, FGFR2, FGFR3, FGFR4, and FGFR5 ( Fig. 1 ). FGFR1–4 have extracellular ligand binding domains and intracellular tyrosine kinase domains, activating downstream signaling pathways upon ligand binding. FGFR5 (FGFRL1) lacks an intracellular kinase domain and its role is not fully understood. 1 2 3 The FGFR family plays a crucial role in cell proliferation, survival, development, metabolism, tissue repair, and dysregulation can contribute to tumor development. 4

## The Biological Basis of the FGFR Gene

FGFR signaling pathway is primarily activated in a ligand-dependent manner through binding to fibroblast growth factor (FGF) ligands. This triggers dimer formation and self-phosphorylation, leading to activation of downstream pathways including RAS-RAF-MAPK, PI3K-AKT, Signal transducer and activator of transcription (STAT), and Phospholipase C γ. This pathway is crucial for normal cell growth, differentiation, neovascularization, proliferation, migration, organ development, and wound healing. 5 Mutations or overexpression of FGFR can result in excessive pathway activation or ligand-independent activation, promoting carcinogenesis. Excessive RAS-RAF-MAPK activation stimulates proliferation and differentiation, PI3K-AKT activation inhibits apoptosis, STAT promotes invasion and metastasis, and PLC γ regulates tumor cell metastasis. FGFR gene abnormalities are common in various cancers, including urothelial, breast, endometrial, and squamous cell carcinomas. 6 7 8 9 10

### Carcinogenic Gene Mutation of FGFR

Oncogenic FGFR pathway activation is primarily caused by dysregulated FGF ligands and abnormal activation mutations in the FGFR gene. These include single-nucleotide variations (SNVs) leading to activation mutations, FGFR gene amplification causing protein overexpression, and FGFR gene fusion mutations resulting in abnormal signaling pathways. 11

FGFR-activated SNVs occur in various domains of FGFR, including the extracellular, transmembrane, and kinase domains. These mutations enhance ligand affinity, receptor dimerization, and ligand-independent activation. Abnormal disulfide bond formation and receptor dimerization in the extracellular domain lead to aberrant receptor signaling, exemplified by C278F mutations in Crouzon and Pfeiffer syndrome, 12 13 as well as C278F and C340F/R/S/W/Y mutations in seminoma. 14 Activation mutations in the transmembrane domain induce receptor rotation, 15 such as Y376C in FGFR2 and G372C, S373C, Y375C, G377C, I378C, Y381C in FGFR3, resulting in ligand-independent receptor activation found in various cancers. 16 17 18 Activation mutations in the kinase domain promote downstream signaling and cancer progression, such as K655I and K656D/E/M/N mutations in FGFR1, 19 K660E/M/N mutation in FGFR2, 20 and K652E/M/N/Q/T mutation in FGFR3. 21 22 These mutations have significant implications for cancer and developmental syndromes. Erdafitinib has been approved for treating urothelial carcinoma patients with FGFR2/FGFR3 mutations, 23 and clinical studies are underway for other solid tumors. 24 25

FGFR gene amplification is the most common FGFR variation in human cancer, accounting for 66% of all FGFR mutations. 8 Amplification leads to FGFR protein overexpression, resulting in abnormal receptor activation and increased downstream signaling. FGFR1 is the most frequently amplified gene, found in approximately 17% of squamous cell carcinoma and 6% of small cell lung cancer, serving as an adverse prognostic marker for early nonsmall cell lung cancer. FGFR1 amplification is also common in breast cancer, with approximately 15% of hormone receptor-positive patients and 5% of triple-negative breast cancer patients exhibiting amplification. FGFR2 amplification is less frequent, occurring in 5 to 10% of gastric cancer (particularly invasive diffuse subtype 2) and 2% of breast cancer, with approximately 4% of triple-negative breast cancer cases showing amplification. FGFR3 and FGFR4 gene amplifications are rare, with frequencies of 0.31 and 0.16% across various tumors. 26 Currently, no approved drugs specifically target FGFR amplification, but clinical trials are underway for lung cancer, gastric cancer, and breast cancer, suggesting potential future treatment targets. 27 28 29

FGFR fusion mutations can be categorized as type I and type II. 29 30 Type I fusion involves chromosomal translocation, resulting in fusion of the kinase domain of FGFR with the oligomerization domain of the fusion partner Type II fusion leads to chimeric transmembrane FGFR. Both fusion types have oncogenic potential by promoting ligand-independent dimerization or abnormal substrate recruitment. FGFR fusion genes have been identified in various tumor types ( Table 1 ). Selective FGFR inhibitors have been approved for FGFR fusion-related tumors, making them the focus of clinical research.

**Table 1:** FGFR fusion partners

Gene	5′ gene	3′ gene	Cancer type	References
FGFR1	FGFR1	TACC1	Glioblastoma	54
	FGFR1	FGFR1	Pilocytic astrocytoma	19
	BAG4	FGFR1	Lung squamous cell carcinoma	55
	ERLIN2	FGFR1	Breast cancer	56
	FN1	FGFR1	Phosphaturic mesenchymal tumor	56
	FOXO1	FGFR1	Rhabdomyosarcoma	57
	SQSTM1	FGFR1	Leukemia	57
FGFR2	FGFR2	AFF3	Breast cancer	56
	FGFR2	CASP7	Breast cancer	56
	FGFR2	CCDC6	Breast cancer	56
	FGFR2	AHCYL1	Cholangiocarcinoma	58
	FGFR2	KIAA1598/SHOOTIN1	Cholangiocarcinoma	59
	FGFR2	MGEA5	Cholangiocarcinoma	45
	FGFR2	PPHLN1	Cholangiocarcinoma	60
	FGFR2	TACC3	Cholangiocarcinoma	59
	FGFR2	BICC1	Colorectal cancer	58
	FGFR2	BICC1	Hepatocellular	58
	FGFR2	CIT	Lung adenocarcinoma	61
	FGFR2	KIAA1967/CCAR2	Lung squamous cell	56
	FGFR2	FAM76A	Ovarian cancer	62
	FGFR2	OFD1	Thyroid cancer	56
FGFR3	FGFR3	TACC3	Bladder cancer	3
	FGFR3	TACC3	Gallbladder cancer	63
	FGFR3	TACC3	Glioblastoma	54
	FGFR3	TACC3	Head and neck squamous cell carcinoma	54
	FGFR3	BAIAP2L1	Lung adenocarcinoma	64
	FGFR3	TACC3	Lung adenocarcinoma	64
	FGFR3	BAIAP2L1	Lung squamous cell carcinoma	64
	FGFR3	TACC3	Lung squamous cell carcinoma	65
	FGFR3	TACC3	Oral cancer	56
	TEL/ETV6	FGFR3	Lymphoma	66
FGFR4	ANO3	FGFR4	Nonsmall cell lung cancer	67
	NSD1	FGFR4	Nonsmall cell lung cancer	67

Abnormal amplification of FGF genes can lead to overexpression of FGF ligands, resulting in excessive activation of downstream carcinogenic signaling pathways. FGF3, FGF4, and FGF19, located on chromosome 11q13, are frequently coamplified in various cancers, including approximately 40% esophageal squamous cell carcinoma, 31 approximately 40% lung squamous cell carcinoma (in smokers), 32 approximately 7% breast cancer, 33 and approximately 4% hepatocellular carcinoma. 34 Patients with FGF3/4/19 amplification mutations have shown benefits from FGFR inhibitors, such as a breast cancer patient with FGF3/4/19 amplification benefiting from pazopanib for over 16 months 35 and two patients with FGF19 amplified hepatocellular carcinoma achieved complete remission after sorafenib treatment. 36 Clinical trials investigating the functional mechanism and clinical use of FGF3/4/19 amplification are strongly recommended.

## Mutation Frequency of FGFR in Various Cancer Species

**Fig. 2:**
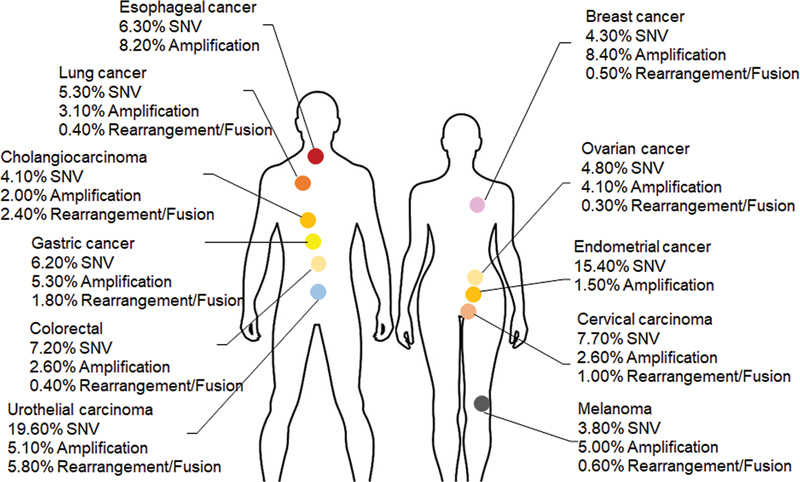
Variation frequency of FGFR in different cancer types. SNV, single-nucleotide mutation.

FGFR mutations are present in almost all malignant tumors. High incidence is observed in urothelial carcinoma, cholangiocarcinoma, breast cancer, endometrial carcinoma, and squamous cell carcinoma. 26 Abnormal FGFR activation is also found in lung cancer, liver cancer, and breast cancer. In a study of 4,853 solid tumor patients, FGFR gene mutations were detected in 7.1% of cases, with frequencies of 3.5% for FGFR1, 1.5% for FGFR2, 2.0% for FGFR3, and 0.5% for FGFR4. Gene amplification was the most common variation (66%), followed by SNV (26%) and rearrangement (8%). Urothelial tumors accounted for 32% of cases, followed by breast cancer at approximately 20%. 26 In a Chinese patient population dataset of 10,194 solid tumors (China Pan-cancer dataset from Cbioportal database), 37 FGFR mutation frequencies were higher than in the Western population, with FGFR1 at 10.68%, FGFR2 at 8.06%, FGFR3 at 5.94%, and FGFR4 at 4.79%. Gene amplification was the main variation form (58.2%), followed by SNV (32.9%) and rearrangement (8.9%). The most common tumor types were urothelial tumors (30.5%) and endometrial cancer (16.9%) ( Fig. 2 ; Table 2 ).

**Table 2:** The mutational frequency of FGFR in China pan-cancer database

Cancer type	Single-nucleotide mutations (%)	Amplification (%)	Rearrangement/fusion (%)	Total (%)
Urothelial carcinoma	19.6	5.1	5.8	30.5
Endometrial cancer	15.4	1.5	0	16.9
Esophageal cancer	6.3	8.2	0	14.5
Gastric cancer	6.2	5.3	1.8	13.3
Breast cancer	4.3	8.4	0.5	13.2
Cervical carcinoma	7.7	2.6	1	11.3
Colorectal	7.2	2.6	0.4	10.2
Melanoma	3.8	5	0.6	9.4
Ovarian cancer	4.8	4.1	0.3	9.2
Lung cancer	5.3	3.1	0.4	8.8
Cholangiocarcinoma	4.1	2	2.4	8.5

## Types of Detection Methods and Their Limitations

FGFR gene activation mutations can be detected through various methods, including SNVs, gene amplification, fusion/rearrangement, and overexpression. Clinical detection methods currently used include next-generation sequencing (NGS), immunohistochemistry (IHC), fluorescence in situ hybridization (FISH), and polymerase chain reaction (PCR). Different methods have limitations, requirements, and performance variations, so appropriate testing methods should be selected based on clinical conditions. Multiplatform testing may be necessary for complementary and validated results.

## Detection of FGFR Single-Nucleotide Mutation

The main methods for detecting FGFR mutations are Sanger sequencing, real-time (RT)-PCR, and NGS. Sanger sequencing can identify known and unknown mutations but requires a high tumor cell content. 38 RT-PCR selectively amplifies FGFR mutations with high sensitivity and specificity. Currently, based on the approval of erdafitinib for the indication of urothelial carcinoma, the FGFR RGQ RT-PCR assay kit (Qiagen) has been approved by the Food and Drug Administration (FDA) for the companion diagnostic testing of FGFR3 point mutations in urothelial carcinoma. However, this technology only allows detection of known mutation sites (FGFR3: p248C, p.G370C, p.S249C, p.Y373C) and cannot detect unknown mutation sites. 25 39 NGS can detect known and unknown variants, including clinically relevant mutations and gene amplification, with high sensitivity and specificity for the FGFR family. NGS is suitable for detecting multiple gene and mutation sites in a single test. 40

## Detection of FGFR Gene Amplification

Both FISH and NGS can detect FGFR amplification. FISH utilizes fluorescent-labeled nucleic acid probes to hybridize with DNA target sequences in the nucleus, and gene copy number is determined by analyzing the fluorescence signal. 41 However, there is significant variation in the standards for determining the FGFR status using FISH. Some clinical studies use an FGFR copy number > 6 as the cutoff value for amplification, whereas others define amplification as FGFR1/CEN8 (centromere of chromosome 8) > 2. Currently, there is no unified standard. 42 43 NGS is more efficient in detecting FGFR amplification mutations due to its ability to simultaneously detect variations in multiple genes. The most commonly used method in NGS for detecting copy number variations (CNVs) in samples is based on read depth analysis. This method involves measuring the sequencing depth of genes or genomic regions to infer CNVs. The steps include sequencing the sample, aligning and mapping the data, and calculating the average sequencing depth for each gene or region. The sample's average sequencing depth is then compared with a reference or control sample to determine CNVs. However, there is currently no universally defined cutoff value for defining amplification variations. FoundationOne defines amplification as a copy number of 4 or higher, serving as a reference standard.

## Detection of FGFR Protein Overexpression

IHC is a standard method for measuring protein overexpression levels. In a study of gastric cancer patients, FGFR2b overexpression was found in 4% of cases, and there was high consistency between IHC and FISH results. 44 However, IHC for FGFR overexpression is not a routine test, and commercially available antibodies are currently lacking. The skills of personnel involved in IHC testing and interpretation can also affect accuracy and repeatability. Criteria for judgment can refer to other protein detection indicators, and IHC scores of 0, 1 + , 2 + , and 3+ can be determined based on cell membrane staining intensity.

## Detection of FGFR Gene Fusion/Rearrangement

FGFR fusion/rearrangement can be detected using PCR, IHC, FISH, NGS, and other methods. FISH and NGS are recommended as clinical detection techniques for FGFR fusion/rearrangement according to guidelines and expert consensus.

### Reverse Transcription-Polymerase Chain Reaction

RT-PCR enables qualitative and quantitative detection of fusion mutations through RNA reverse transcription. It is cost-effective and highly sensitive and specific. However, it can only detect known fusion forms and has low throughput. Different fusions require separate detection, making it unsuitable for genes with multiple targets in the FGFR family. The FGFR RGQ RT-PCR kit (Qiagen) is FDA-approved for detecting FGFR2/FGFR3 fusion variants in urothelial carcinoma (FGFR2-BICC1, FGFR2-CASP7, FGFR3-TACC3, FGFR3-BAIAP2L1).

#### Immunohistochemistry

IHC detects fusion proteins using specific antibodies. It requires a small sample size and can be done with one formalin-fixed, paraffin-embedded (FFPE) slide. However, IHC has limitations in FGFR fusion detection. It has low sensitivity for rare fusions and cannot determine fusion partners or subtypes. No IHC method has sufficient sensitivity and specificity for FGFR fusion detection. Antibodies may have similar epitopes on different targets, leading to false positives in FGFR fusion detection.

### Fluorescence In Situ Hybridization

FISH is a widely used clinical testing method for detecting fusion in various cancers, considered the gold standard for fusion detection. It requires a small amount of tissue, is cost-effective, and can detect fusion within target cells. However, FISH has limitations in determining specific fusion genes and breakpoints, and complex rearrangements may be missed. Literature reports indicate that chromosomal rearrangements contribute to around 50% of FGFR2 fusions in intrahepatic cholangiocarcinoma (iCCA), with the possibility of false negatives in FISH analysis. 45 Furthermore, due to its low throughput and limited ability to detect only one target at a time, FISH analysis is time-consuming for the detection of multiple genes, such as FGFR1–4.

### Next-Generation Sequencing

NGS provides an accurate and efficient method for detecting fusion. It can detect multiple fusion forms in a single tumor sample, with lower overall time and cost compared with IHC and FISH. NGS is particularly suitable for detecting multiple gene and fusion forms in the FGFR family. Dual testing of DNA and RNA levels is recommended for accurate fusion detection.

#### DNA-Based Next-Generation Sequencing

NGS analysis strategies for DNA-based detection include whole-genome sequencing (WGS), whole-exome sequencing (WES), and targeted sequencing. WGS can identify a large number of rearrangements and breakpoints, including noncoding regions, making it effective for discovering new fusion mutants. However, WGS is expensive and time-consuming due to the large amount of data and computational analysis. WES has a lower cost but is less suitable for detecting fusion mutations, as it only covers exon region breakpoints. Targeted sequencing is a cost-effective method for accurately detecting exon and intron region breakpoints, but it loses other genomic information.

#### RNA-Based Next-Generation Sequencing

RNA-based detection is more sensitive and efficient for fusion mutations compared with DNA-based detection. RNA-based methods can distinguish between intraframe and interframe fusion and avoid sequencing large intron regions. However, sensitivity depends on fusion expression levels, and RNA is less stable than DNA, especially in FFPE samples, which may lead to errors due to sample deterioration or degradation. 46

## Testing Requirements of FGFR

### Content Requirements for FGFR Testing Report

The NGS or RT-PCR report for FGFR detection should include (1) patient information: name, gender, age, outpatient/inpatient ID, physician's name, and clinical indications; (2) sample information: type, collection date and location, identification number, submission and report generation dates, tumor cell content, DNA quality, sequencing quality, etc.; (3) detection details: instruments, reagents, methods, panel coverage, detection limit, etc.; (4) test results and explanations: genotype, mutation details, relevant drug information, supporting evidence for each mutation, and limitations of the experiment.

The FISH report for FGFR detection should include: patient information (name, gender, age, outpatient/inpatient ID), physician's name, submission date, pathological report ID, sample collection location, specimen type, probe information, detection method, use of image analysis, control settings, sample size sufficiency, results explanation (cell count, average FGFR copy count/cell, ratio of FGFR copy count/FGFR centromere copy count), and test results (positive, negative, IHC validation required, uncertainty).

The IHC report for FGFR testing should include: patient information (name, gender, age, outpatient/inpatient ID), physician's name, submission date, pathological report ID, sample collection location, sample type, antibody information, testing method, use of image analysis, control settings, sample size sufficiency, and result interpretation (0, 1 + , 2 + , 3 + ).

### Detection Process of FGFR

Different FGFR detection methods have varying sensitivity, specificity, advantages, and limitations. Doctors should choose an appropriate testing platform based on specimen type, sample size, tumor cell content, sample quality, clinical needs, and laboratory capabilities. Simultaneous testing on multiple platforms is recommended for result accuracy.

For efficient and accurate FGFR mutation detection, NGS is recommended to detect multiple mutation forms simultaneously. RT-PCR can be used as an alternative for SNVs and small insertions/deletions, whereas FISH can detect amplification and fusion. IHC can be used for protein overexpression. FISH and IHC are limited to tissue samples, whereas NGS and PCR can be applied to circulating tumor DNA (ctDNA) samples.

## Quality Control of FGFR Detection

### Selection of Sample and Methods for Processing

To preserve tumor tissue specimens, it is important to obtain as many diagnostic specimens as possible at once. NGS multigene testing can provide more genetic information from limited samples, minimizing the need for invasive sampling and guiding treatment decisions. Tumor tissue and cytological samples should be evaluated for tumor cell content. Consideration should be given to the laboratory environment, sample type, size, and quality control (QC) results when selecting a testing method. Liquid biopsy specimens can be used as supplementary tests, but limitations should be clearly stated in the report.

### Samples of Tumor Tissue

Tumor tissue samples, including fresh tissue and FFPE samples, should be evaluated for tumor cell content before FGFR testing. A minimum tumor cell content of 20% is recommended. If the content is lower, tumor cell enrichment is advised, with sample limitations clearly stated in the report.

FFPE specimens should be processed according to pathological specifications. Tumor tissue should be fixed with neutral-buffered formalin promptly after separation or removal from liquid nitrogen. Surgical tissue requires 12 to 48 hours of fixation (not exceeding 72 hours), whereas biopsy tissue requires 6 to 12 hours. FFPE samples should not be stored for more than 24 months.

### Liquid Biopsy

For patients without tumor tissue, ctDNA enrichment from blood, urine, cerebrospinal fluid, pleural, and abdominal fluid samples can be used for detection. CtDNA, released by tumor cells into body fluids, can provide genomic variation information detectable through DNA-based NGS, including SNV, amplification, and fusion. CtDNA detection is widely used due to its simplicity, extracting, and sequencing DNA from blood samples. However, the sensitivity of ctDNA detection is relatively low for larger structural variations, limiting its accuracy. Collection, preservation, and transportation of ctDNA: STRECK Blood collection tubes are suitable for collecting and preserving ctDNA in blood. Blood collection should be stored at 6 to 37°C without freezing or thawing. Unused tubes should be stored at 2 to 30°C. For transportation, use constant temperature (15–25°C) if temperatures are below 6°C or above 30°C; otherwise, normal temperature transportation is sufficient. If necessary, samples can be stored overnight at 6 to 37°C. CtDNA in whole blood can be stored for 3 to 7 days at room temperature.

For urine collection, use a self-prepared urine cup and connect it to a urine filter and syringe sleeve. Pour 40 to 120mL of urine into the syringe sleeve, allowing it to pass through the filter and collect in the yellow cap urine storage cup. Urine should be stored and transported at room temperature. Transferred urine can be stored for 30 days at room temperature.

## Treatments for FGFR-Altered Solid Tumors

Approved drugs for FGFR can be categorized into multitarget FGFR inhibitors and selective FGFR inhibitors.

### Multitarget FGFR Inhibitors

Multitarget FGFR inhibitors, the first generation FGFR inhibitors, have low selectivity and also target vascular endothelial growth factor receptor (VEGFR) and platelet-derived growth factor receptor (PDGFR). Several drugs have been approved for cancer treatment: sorafenib for advanced renal cell carcinoma, liver cancer, and thyroid cancer; sunitinib for gastrointestinal stromal tumors, renal cell carcinoma, and pancreatic neuroendocrine tumors; regorafenib for colorectal cancer, gastrointestinal stromal tumor, thyroid cancer, and liver cancer; pazopanib for renal cell carcinoma and sarcoma; lenvatinib for thyroid cancer and hepatocellular carcinoma.

Multitarget FGFR inhibitors have broad activity against various cancer-related receptors but lack selectivity. Their anticancer properties depend on VEGFR and PDGFR inhibition, reducing the effective therapeutic concentration for FGFR inhibition. They have high systemic toxicity and adverse reactions, including hypertension, fatigue, and gastrointestinal issues, limiting their clinical use.

### Selective FGFR Inhibitors

Selective FGFR inhibitors can be categorized as noncovalent and covalent inhibitors. Noncovalent selective FGFR inhibitors, such as erdafitinib, pemigatinib, infigratinib, and AZD4547, have been developed to address the systemic toxicity of multitarget FGFR inhibitors ( Table 3 ). FDA has approved erdafitinib, pemigatinib, and infigratinib for cancer treatment.

**Table 3:** Selective FGFR inhibitors in clinical development

Drug	Targets	Cancer	Phase of ongoing trials
Erdafitinib	FGFR1–4	Urothelial carcinoma/bladder cancer/lung cancer/prostate carcinoma/solid tumor	III/II/II/II/II
Infigratinib	FGFR1–3	Cholangiocarcinoma/urothelial carcinomas/gastric cancer/central nervous system tumor/solid tumor	III/III/II/II/II
Pemigatinib	FGFR1–3	Cholangiocarcinoma/urothelial cancer/bladder cancer/nonsmall cell lung cancer/gastric and colorectal cancer/breast cancer/solid tumor	III/II/II/II/II/II/II
Futibatinib	FGFR1–4	Cholangiocarcinoma/breast cancer/endometrial carcinoma/urothelial cancer/pediatric cancer/solid tumor	II/II/II/II/II/II
Gunagratinib	FGFR1–4	Intrahepatic cholangiocarcinoma/urothelial carcinoma	II/II
Rogaratinib	FGFR1–3	Urothelial carcinoma/transitional cell	III/III
Derazantinib	FGFR1–4	Cholangiocarcinoma/urothelial carcinoma/gastric cancer/solid tumor	II/II/II/II
AZD4547	FGFR1–3	Urothelial carcinoma/gliomas/nonsmall cell lung cancer/breast cancer/gastric cancer/esophageal cancer/solid tumor	II/II/II/II/II/II/II

Erdafitinib is a noncovalent FGFR1–4 inhibitor approved by the FDA for locally advanced or metastatic urothelial carcinoma patients with FGFR2/FGFR3 mutations after platinum-based chemotherapy. Clinical trial results showed an objective response rate (ORR) of 40%, median progression-free survival (mPFS) of 5.5 months, and median overall survival (OS) of 13.8 months. Common adverse reactions include hyperphosphatemia, fatigue, dry mouth, eye adverse reactions, nail adverse reactions, constipation, and anorexia. Eye adverse reactions occur in approximately 28% of patients, and the FDA recommends dry eye prevention and regular eye examinations. 47

Pemigatinib is a noncovalent FGFR1–3 inhibitor approved by the FDA for locally advanced, recurrent, or metastatic cholangiocarcinoma with FGFR2 fusion or rearrangement after systemic treatment failure. Clinical trial results showed an ORR of 35.5%, mPFS of 6.9 months, median duration of response (DOR) of 7.5 months, and median disease control rate (DCR) of 82.0%. Common adverse reactions include hyperphosphatemia, stomatitis, joint pain, and hyponatremia. Approximately 19% of patients stopped treatment due to adverse reactions. 48 Furthermore, based on data presented at the 2023 American Association for Cancer Research, the clinical study (FIGHT-207) evaluating pemigatinib in patients with nonresectable, advanced/metastatic solid tumors harboring FGFR mutation/fusion demonstrated an ORR of 26.5% (95% confidence interval [CI]: 15.0–41.1%) and a DCR of 65.3% (95% CI: 50.4–78.3%). 49 These findings indicate that pemigatinib exhibits antitumor activity across various cancer types.

Infigratinib is a selective noncovalent FGFR1–3 inhibitor approved by the FDA for previously treated, unresectable locally advanced or metastatic cholangiocarcinoma with FGFR2 fusion or rearrangement. Clinical trial results showed an ORR of 23% and a DOR of 5.0 months. Common adverse reactions include hyperphosphatemia, stomatitis, fatigue, hair loss, and dry eye syndrome. Regular ophthalmic examinations are important during infigratinib treatment. 50

Futibatinib is a selective covalent FGFR1–4 inhibitor approved by the FDA for unresectable, locally advanced, or metastatic iCCA with FGFR2 gene fusion or rearrangements. Phase II trial results showed an ORR of 42%, median time for maintaining efficacy of 9.7 months, median time without disease progression of 9.0 months, and median OS time of 21.0 months. Common adverse reactions include hyperphosphatemia, diarrhea, dry mouth, and dry skin. 51

Gunagratinib is a selective covalent FGFR1–4 inhibitor approved by the FDA for cholangiocarcinoma patients who have received first-line systemic chemotherapy and have FGFR2 heterotopic or fused. Clinical data from a phase IIA dose extension study showed an ORR of 52.9%, DCR of 94.1%, and mPFS of 6.93 months. Gunagratinib demonstrated a higher response rate and good safety and tolerability compared with other approved FGFR inhibitors. 52

Ongoing clinical trials are evaluating other selective noncovalent FGFR inhibitors, including AZD4547 and Debio-1347. Selective FGFR inhibitors offer improved targeting and reduced adverse reactions compared with nonselective inhibitors. However, adverse reactions, particularly hyperphosphatemia and tissue calcification resulting from FGFR pathway regulation, still limit their clinical use. Acquired drug resistance remains a major challenge for selective noncovalent FGFR inhibitors. 53

## Summary and Prospect

**Fig. 3:**
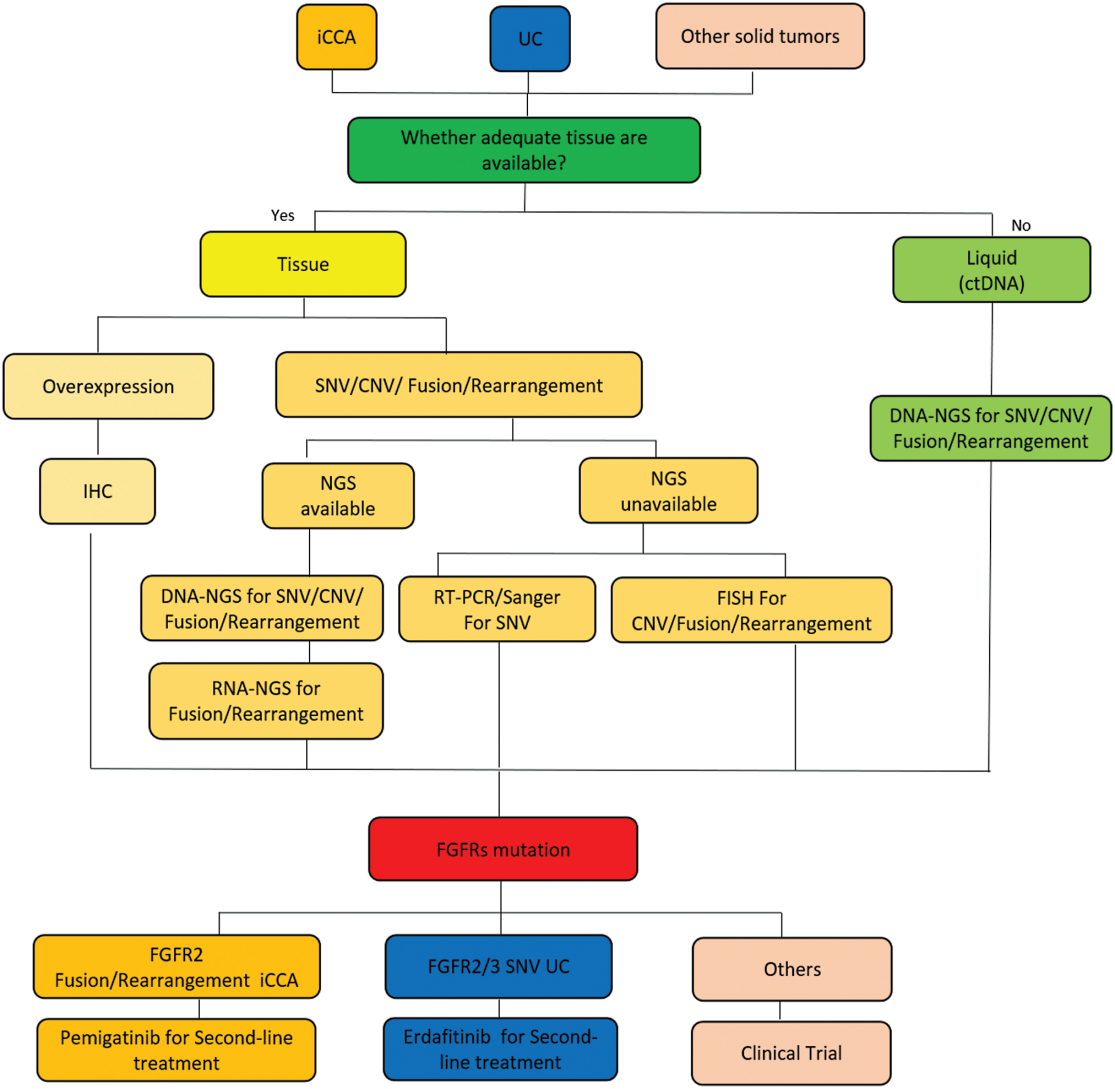
Testing process of FGFR. CNV, copy number variation; ctDNA, circulating tumor DNA; FISH, fluorescence in situ hybridization; iCCA, intrahepatic cholangiocarcinoma; IHC, immunohistochemistry; NGS, next-generation sequencing; RT-PCR, real-time polymerase chain reaction; SNV, single-nucleotide mutation; UC, urothelial cancer.

Based on current evidence, clinical guidelines from organizations like the National Cancer Comprehensive Network and the Chinese Society of Clinical Oncology recommend FGFR mutation testing for clinical diagnosis, treatment, and clinical trials. FGFR has emerged as a prominent target in “unlimited cancer” treatment due to its high mutation frequency across various tumor types. The development of more approved drugs for different tumor types is anticipated. To address this, we have established expert consensus and practical guidelines for managing FGFR-related tumors ( Table 4 ; Fig. 3 ). These guidelines encompass the prevalence of different FGFR mutations (including mutation, amplification, overexpression, fusion/rearrangement), detection methods, QC standards, testing report requirements, and treatment plans. Dissemination and implementation of this consensus are of significant clinical importance. However, the recommended testing strategy may be influenced by regulatory policies in the health inspection field. Experts are encouraged to actively utilize this consensus, industry standards, and guidelines to advocate for favorable policies. Limited published research in China has resulted in most of the cited studies originating from abroad, which somewhat restricts the evidence-based application of this consensus in China. Furthermore, certain aspects of the consensus, particularly those related to QC, lack supporting evidence from large-scale clinical trials. Therefore, further research is necessary to validate the information provided in this consensus.

**Table 4:** Expert consensus on the diagnosis and treatment of FGFR gene-altered solid tumors in China

	Consensus number	Key points	Recommendation level
Detection time point	Consensus 1	FGFR mutation detection is recommended for approved tumor types with FGFR selective inhibitors, such as urothelial carcinoma and cholangiocarcinoma	Strongly recommended
	Consensus 2	Pan solid tumors should also undergo FGFR mutation detection, with participation in relevant clinical trials recommended for patients with single-nucleotide mutations, amplifications, or fusion/rearrangements	Recommended
	Consensus 3	NGS is the preferred method for FGFR mutation detection, targeting FGFR1/2/3/4 and including single-nucleotide mutations, amplifications, and fusion/rearrangements	Recommended
Detection strategy	Consensus 4	FGFR testing should prioritize tumor tissue samples (surgery, biopsy, etc.). If tumor tissue is not obtainable, liquid sample testing (blood, urine, effusions, lavage fluid, cerebrospinal fluid, etc.) based on tumor type is recommended. However, the limitations of standard testing should be clearly stated in the report	Strongly recommended
	Consensus 5	ctDNA testing is recommended for patients with difficulty obtaining tissue samples, providing genomic data for targeted treatment and prognosis with high detection sensitivity	Recommended
Detection method	Consensus 6	DNA–NGS panel or WES can be used for gene fusion detection, while RNA–NGS panel or WTS is a supplementary method. Simultaneous extraction of DNA and RNA from fresh tissue or FFPE slices is recommended for NGS detection	Strongly recommended
	Consensus 7	Reagent kits for NGS detection should clearly indicate probe coverage, including introns, to avoid false negatives	Strongly recommended
	Consensus 8	Alternative methods such as RT-PCR and Sanger sequencing can be used for single-nucleotide mutations, and FISH and RT-PCR for amplifications and fusion/rearrangements, when NGS is not feasible	Recommended
	Consensus 9	NGS can be used as a reconfirmation method for patients with unknown fusion or negative test results	Recommended
	Consensus 10	FGFR protein expression can be detected by IHC	Not recommended
Detecting quality control	Consensus 11	Experienced pathologists should evaluate tumor cell content in tissue and cytological samples. At least 50 tumor cells are required for IHC or FISH, 5% for RT-PCR, and 20% for NGS. Microscopic dissection can be considered to enrich tumor tissue if cell content is insufficient	Strongly recommended
	Consensus 12	Laboratories should participate in quality control plans (PQCC, CAP, CLIA, etc.) and compare results with qualified laboratories using the same method. Alternative methods should be available for validation and review of inconsistent results	Strongly recommended
	Consensus 13	Test reports should include basic information, quality control data, tumor cell content, microscopic anatomy status, and DNA concentration and purity. NGS reports should provide details on fusion breakpoints, involvement of tyrosine kinase domains, and intraframe fusion. Fusion involving the tyrosine kinase domain and in-frame fusion should be reported as fusion, while others as rearrangement	Recommended
	Consensus 14	For complex cases or doubts (inconsistent results, new fusion patterns, multidriver genes, etc.), consultation with the Molecular Oncology Board (MTB) is recommended for treatment decisions	Strongly recommended
Treatment strategy	Consensus 15	Erdafitinib should be considered for FGFR2/3 variant urothelial carcinoma treatment (FDA-approved for locally advanced or metastatic urothelial carcinoma after platinum-based chemotherapy, undergoing NMPA listing in China)	Strongly recommended
	Consensus 16	For FGFR2 fusion/rearrangement in intrahepatic cholangiocarcinoma, consider treatment with pemigatinib, infigratinib, futibatinib, gunagratinib, etc. (FDA-approved for posterior treatment of unresectable, locally advanced, or metastatic intrahepatic cholangiocarcinoma, with pemigatinib approved for domestic marketing by NMPA)	Strongly recommended
	Consensus 17	For FGFR mutation patients with unapproved tumor types or mutation types, participation in relevant clinical trials is recommended	Recommended

Abbreviations: CAP, College of American Pathologists; CLIA, Clinical Laboratory Improvement Amendments; ctDNA; circulating tumor DNA; FDA, Food and Drug Administration; FFPE, formalin-fixed, paraffin-embedded; FISH, fluorescence in situ hybridization; IHC, immunohistochemistry; NGS, next-generation sequencing; NMPA, National Medical Products Administration; PQCC, Pathology Quality Control Center; RT-PCR, real-time polymerase chain reaction; WES, whole-exome sequencing; WTS, whole-transcriptome sequencing.
